# Fractional exhaled nitric oxide and the response to prednisolone for asthma attacks in patients treated with anti-IL5/5Rα therapy: a prospective observational study

**DOI:** 10.1183/13993003.01229-2025

**Published:** 2025-11-06

**Authors:** Imran Howell, Mahdi Mahdi, Hafiz R. Mahmood, Laura Bermejo-Sanchez, Catherine Borg, Sanjay Ramakrishnan, James Melhorn, Gabriel Lavoie, Nayia Petousi, Timothy S.C. Hinks, Mona Bafadhel, Ian D. Pavord

**Affiliations:** 1 Respiratory Medicine Unit and Oxford Respiratory NIHR BRC, Nuffield Department of Medicine, University of Oxford, Oxford, UK; 2 Institute for Respiratory Health and UWA Medical School, University of Western Australia, Perth, Australia; 3 Département de médecine, Université de Montréal, Québec, QC, Canada; 4 King's Centre for Lung Health, School of Immunology and Microbial Sciences, King's College London, London, UK

## Abstract

Anti-interleukin (IL)5/5Rα monoclonal antibody (mAb) therapies deplete blood eosinophils and reduce the annualised asthma attack rate by over 50% [1]. The clinical benefits of oral corticosteroids (OCS) to treat breakthrough attacks are uncertain. Fractional exhaled nitric oxide (*F*_ENO_) has the potential to discriminate between ongoing type-2 airway inflammation or infection in breakthrough attacks on mepolizumab [2].

## Shareable abstract

***F*_ENO_ testing at attack can identify the patients on anti-IL5/IL5Rα biologics who have the most lung function and symptom benefits from prednisolone** https://bit.ly/3KhMB68

*To the Editor:*

Anti-interleukin (IL)5/5Rα monoclonal antibody (mAb) therapies deplete blood eosinophils and reduce the annualised asthma attack rate by over 50% [1]. The clinical benefits of oral corticosteroids (OCS) to treat breakthrough attacks are uncertain. Fractional exhaled nitric oxide (*F*_ENO_) has the potential to discriminate between ongoing type-2 airway inflammation or infection in breakthrough attacks on mepolizumab [2].

We designed a prospective, observational study to investigate the relationship between *F*_ENO_ and the clinical responses to prednisolone treatment for outpatient attacks in anti-IL5/5Rα-treated patients.

A peer-reviewed protocol for the BreakthrOugh asthma attacks treated with Oral STeroids (BOOST) study was published with a pre-specified statistical analysis plan [3]. BOOST was a sub-study of the Oxford Airways Study conducted in the outpatient Oxford specialist asthma service (Oxfordshire Research Ethics Committee reference: 18/SC/0361). Participants provided written informed consent to the Oxford Airways Study.

The inclusion criteria were adults aged 18 years or over treated with anti-IL5 or anti-IL5Rα mAb therapy for at least 2 months prescribed according to UK guidelines for severe eosinophilic asthma. An asthma attack was defined as ≥48 h respiratory symptom deterioration despite increased inhaler usage [4] and no alternative diagnosis after clinical history, physical examination and chest radiograph. The exclusion criteria were treatment with OCS in the 4 weeks prior to the attack visit; pregnancy; systemic immunosuppressive treatment (non-asthma); participant requiring hospitalisation.

Eligible participants were treated with 40 mg of oral prednisolone once daily for 7 days as per usual care [5]. Antibiotic therapy initiated prior to the attack visit was completed. Participants could have treatment for one asthma attack in the study.

Study visits were at stable state (>8 weeks from an attack), attack, and 7 and 28 days after attack. At each visit, participants underwent post-bronchodilator spirometry, *F*_ENO_ (Circassia), physical examination, vital signs, medication check, full blood count, serum C-reactive protein (CRP), sputum differential cell count [6], and the Asthma Control Questionnaire-5 (ACQ-5). Visual analogue scale (VAS) scores for respiratory symptoms were completed electronically at each visit and daily from attack until day 28. At the attack visit a throat swab for multiplex viral PCR (BioFire Diagnostics), sputum for bacterial culture and a neutrophil elastase test (NEATstik, ProAxsis) were carried out.

Comparison of all outcomes were between *F*_ENO_-high (≥25 ppb) and *F*_ENO_-low (<25 ppb) groups defined at attack. The *F*_ENO_-low threshold was based on earlier studies showing a low likelihood of corticosteroid-responsive eosinophilic airway inflammation in patients with a *F*_ENO_ <25 ppb [2, 7].

The primary outcome was the proportion of treatment failure (unscheduled healthcare visit for asthma or repeated acute asthma oral treatment) at day 28 between *F*_ENO_-high and *F*_ENO_-low groups. Treatment failure was self-reported by patients and corroborated with health records. Secondary outcomes were changes in forced expiratory volume in 1 s (FEV_1_), ACQ-5 score, and total VAS symptoms at day 7.

Descriptive statistics categorised by *F*_ENO_ groups are presented. The proportion of treatment failure at day 28 between *F*_ENO_ groups was calculated by Fisher's exact test and time to treatment failure by Kaplan–Meier analysis and stratified log-rank test. Linear mixed effects models compared continuous data from the secondary outcomes. The random effect was the participants, and fixed effects were the study visit, *F*_ENO_ group, and their interaction. The model managed missing data using the missing at random assumption.

BOOST was a pilot study to support designing definitive placebo-controlled trials. The sample size of 60 asthma attacks was based on an anticipated treatment failure rate of 20% at 28 days [8, 9], with 30% absolute difference between *F*_ENO_ groups, two-sided α=0.05, β=0.8, and a 10% dropout.

Between September 2022 until April 2024, 111 patients established on anti-IL5/5Rα treatment with acute respiratory symptoms were assessed for study inclusion. 51 patients were excluded: 23 clinically did not have an asthma attack, and 28 had an asthma attack but met exclusion criteria. 60 were enrolled and treated with open label prednisolone. 21 participants were *F*_ENO_-low (<25 ppb), and 39 participants were *F*_ENO_-high (≥25 ppb) at attack.

In all participants, the mean age was 56 years (range 22–78 years), 63% were female, 85% were of white ethnicity, mean body mass index was 29.6 kg·m^−2^, 23% had chronic rhinosinusitis/nasal polyps, 72% had never smoked and there were no current smokers. These characteristics were similar between *F*_ENO_-low and *F*_ENO_-high groups. The *F*_ENO_-low group had more years with asthma (28 *versus* 16 years; p=0.04). Routine medications were similar between groups. All participants were treated with an inhaled corticosteroid-containing inhaler at a mean 1680 μg beclomethasone dipropionate equivalent dose. 45% were on an antimuscarinic inhaler and 3% on prophylactic azithromycin. Median duration on anti-IL5/5Rα biologic was 23 months (interquartile range 7–43 months). More *F*_ENO_-low participants were on an anti-IL5Rα biologic (62% *versus* 33%; p=0.03). *F*_ENO_-low and -high groups had similar stable state FEV_1_ (2.34 *versus* 2.23 L), ACQ-5 (1.61 *versus* 2.09) and total VAS (13.8 *versus* 21.9 mm). *F*_ENO_-low attacks had quicker symptom deterioration before attack (5 *versus* 10 days; p=0.02), associated fever (43 *versus* 18%; p=0.04), and viral positivity (57 *versus* 29%; p=0.03) compared to *F*_ENO_-high attacks. Sputum culture positivity at attack (20% *versus* 8%) and antibiotic prescription in the 2 weeks before attack (9% *versus* 8%) were similar in *F*_ENO_-low and *F*_ENO_-high groups. Median blood (0.00 *versus* 0.04×10^9^ L^−1^) and sputum (0.0 *versus* 0.6%) eosinophil counts at attack were low in *F*_ENO_-low and *F*_ENO_-high groups at attack. No participant had blood eosinophils ≥0.3×10^9^ L^−1^ at any visit. Median attack *F*_ENO_ was lower in the *F*_ENO_-low group (12 *versus* 69 ppb; p<0.001). Sputum neutrophils (42% *versus* 68%), NEATstik positivity (29% *versus* 44%), median CRP (7 *versus* 5 mg·L^−1^), and median IgE (97 *versus* 167 KU·L^−1^) were similar in *F*_ENO_-low and *F*_ENO_-high groups at attack.

All 60 patients were followed up. There was no significant difference in proportion of treatment failure at day 28 between the *F*_ENO_-low (n=9, 43%) *versus* the *F*_ENO_-high (n=11, 28%) group (OR 1.89, 95% CI 0.54 to 6.64; p=0.27). The time to first treatment failure within 28 days was not significant between the *F*_ENO_-low *versus* the *F*_ENO_-high group (HR 1.87, 95% CI 0.77 to 4.52; log-rank p=0.2).

Between the attack and day 7, the *F*_ENO_-high group had significantly greater improvements in their FEV_1_ (373 mL *versus* 3 mL, mean difference 370 mL, 95% CI 113 to 628 mL; p=0.006), ACQ-5 (−1.68 *versus* −0.27, mean difference −1.41, 95% CI −0.70 to −2.11; p<0.001), and total VAS symptoms (−25.9 *versus* −12.9, mean difference −13.0, 95% CI −4.2 to −21.9; p<0.005) compared to the *F*_ENO_-low group (figure 1).

**FIGURE 1:**
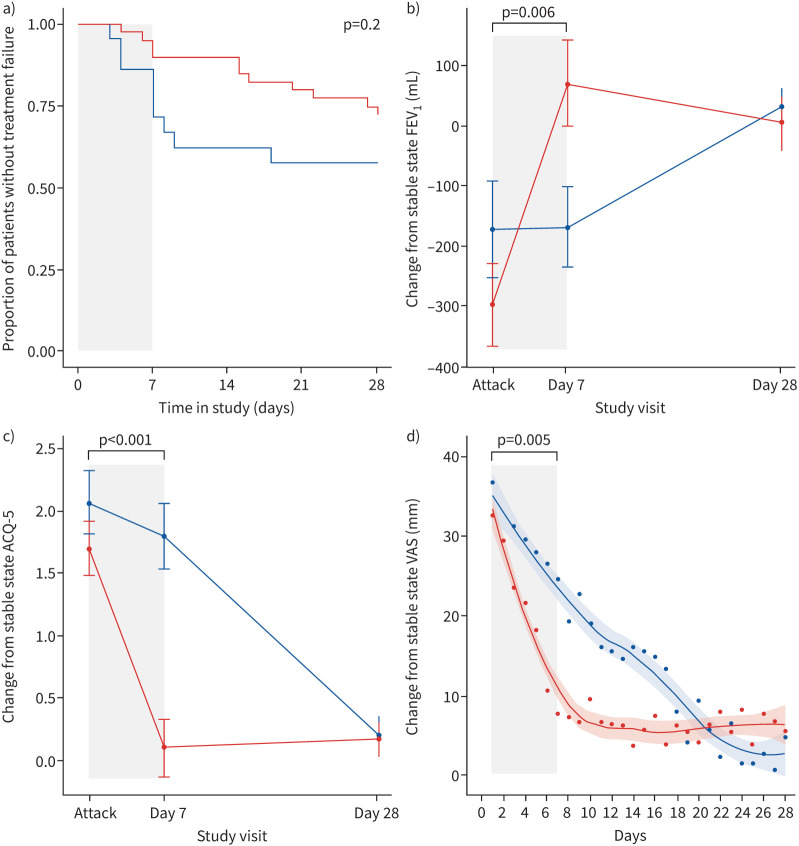
Comparison of clinical outcomes between fractional exhaled nitric oxide (*F*_ENO_)-high and *F*_ENO_-low groups from attack to day 28. a) Kaplan–Meier plot of time to first treatment failure; b) forced expiratory volume in 1 s (FEV_1_); c) Asthma Control Questionnaire-5 (ACQ-5); d) total visual analogue scale (VAS) symptoms. The red line indicates the *F*_ENO_-high group, and the blue line indicates the *F*_ENO_-low group. The vertical grey shaded area indicates when prednisolone treatment was given between the attack and day 7 visits. In plots b–d, the 0 value on the *y*-axis corresponds to stable state. All mean values, and the mean difference in change from attack to day 7 between *F*_ENO_-high and *F*_ENO_-low groups, were calculated by linear mixed effects models. The curves in plot d were fit by LOESS regression.

In multivariable regression, after adjusting for biologic class, sex, asthma duration (continuous), stable FEV_1_ (continuous), stable ACQ-5 (continuous), viral status, attack sputum eosinophil percentage (continuous), and attack blood eosinophil count (continuous), only attack *F*_ENO_ (continuous) was associated with a significant improvement in FEV_1_ (L) (0.004 per ppb, 95% CI 0.001 to 0.007; p=0.02) and ACQ-5 (−0.01 per ppb, 95% CI −0.003 to −0.018; p=0.005) between attack and day 7.

The BOOST study showed no statistical difference in rate of treatment failure between *F*_ENO_ groups at day 28. This result likely reflects a small study sample size and more late treatment failures in the *F*_ENO_-high group than anticipated. Nonetheless, we demonstrated that *F*_ENO_-high attacks were associated with more rapid and larger symptom and lung function improvement after 7 days prednisolone treatment compared to *F*_ENO_-low attacks in a population where blood eosinophils were suppressed by anti-IL-5/5Rα treatment.

Raised *F*_ENO_ at attack has been associated with sputum eosinophilia in an anti-IL5-treated population [2]. In BOOST this was not seen, potentially reflecting greater suppression of blood eosinophilia in patients treated with benralizumab [10]. The relationship between *F*_ENO_ with symptom and lung function recovery after OCS treatment was independent of sputum eosinophils. This questions whether sputum eosinophils alone predict OCS responsiveness and suggests involvement of broader, active steroid-responsive inflammation [11]. Broader suppression of type-2 inflammation may be beneficial in patients with ongoing attacks, despite anti-IL5/5Rα treatment.

*F*_ENO_-low attacks were associated with viral infection and displayed small, non-clinically meaningful improvements to symptoms and lung function after OCS treatment. This is consistent with a prior study conducted in a mAb-naive population [12]. Together, these studies question the value of OCS treatment for blood eosinophil and *F*_ENO_ suppressed outpatient asthma attacks. The decision to prescribe medical treatment is contingent on its risk–benefit balance, and OCS have a significant risk of side-effects [13]. Biomarker-directed, placebo-controlled trials of OCS treatment for outpatient asthma attacks are required to re-evaluate whether using OCS for type-2 low attacks is clinically effective. Since symptoms and lung function declined similarly at attack in both *F*_ENO_ groups, alternative treatable mechanisms need to be explored in the type-2 low population [14, 15].

The strengths of the BOOST study are the prospective design with a predefined *F*_ENO_ threshold for analysis based on a low risk of steroid responsiveness below 25 ppb [7], and the detailed serial assessment of clinical and airway inflammatory variables around a time of great clinical interest. The main limitations are that this was a small, single-centre, unblinded and non-randomised study. There was a risk of confounding due to differing baseline characteristics between *F*_ENO_ groups. The conclusions of this work are preliminary and need to be confirmed in definitive, larger trials.

In conclusion, the BOOST study demonstrates that *F*_ENO_ testing at attack can identify the patients on anti-IL5/IL5Rα treatment who have the most lung function and symptom benefit from prednisolone.

## Shareable PDF

10.1183/13993003.01229-2025.Shareable1 This PDF extract can be shared freely online.
Shareable PDF

ERJ-01229-2025.Shareable

## Data Availability

Deidentified participant data and code scripts are available on request after publication.
