# Supplementary material for: Fractional exhaled nitric oxide and the response to prednisolone for asthma attacks in patients treated with anti-IL5/5Rα therapy: a prospective observational study
Source: Eur Respir J. 2025 Nov 6;66(5):2501229. doi: 10.1183/13993003.01229-2025 (PMC12591134; doi:10.1183/13993003.01229-2025)
Supplement: Supplementary file 1 [file ERJ-01229-2025.Shareable.pdf]

EUROPEAN RESPIRATORY JOURNAL
RESEARCH LETTER
I. HOWELL ET AL.

SHAREABLE PDF

Fractional exhaled nitric oxide and the response to prednisolone for asthma attacks in patients treated with anti-IL5/5Rα therapy: a prospective observational study

Imran Howell [1], Mahdi Mahdi[1], Hafiz R. Mahmood[1], Laura Bermejo-Sanchez[1], Catherine Borg[1], Sanjay Ramakrishnan [2], James Melhorn [1], Gabriel Lavoie[3], Nayia Petousi [1], Timothy S.C. Hinks [1], Mona Bafadhel [4] and Ian D. Pavord[1]

[1]Respiratory Medicine Unit and Oxford Respiratory NIHR BRC, Nuffield Department of Medicine, University of Oxford, Oxford, UK. [2]Institute for Respiratory Health and UWA Medical School, University of Western Australia, Perth, Australia. [3]Département de médecine, Université de Montréal, Québec, QC, Canada. [4]King's Centre for Lung Health, School of Immunology and Microbial Sciences, King's College London, London, UK.

Corresponding author: Imran Howell (imran.howell@ndm.ox.ac.uk)

Shareable abstract (@ERSpublications)
F_{ENO} testing at attack can identify the patients on anti-IL5/IL5Rα biologics who have the most lung function and symptom benefits from prednisolone https://bit.ly/3KhMB68

Cite this article as: Howell I, Mahdi M, Mahmood HR, *et al*. Fractional exhaled nitric oxide and the response to prednisolone for asthma attacks in patients treated with anti-IL5/5Rα therapy: a prospective observational study. *Eur Respir J* 2025; 66: 2501229 [DOI: 10.1183/13993003.01229-2025].

This PDF extract can be shared freely online.

Copyright ©The authors 2025

This version is distributed under the terms of the Creative Commons Attribution Licence 4.0.

Received: 18 March 2025
Accepted: 5 Sept 2025

To the Editor:

Anti-interleukin (IL)5/5Rα monoclonal antibody (mAb) therapies deplete blood eosinophils and reduce the annualised asthma attack rate by over 50% [1]. The clinical benefits of oral corticosteroids (OCS) to treat breakthrough attacks are uncertain. Fractional exhaled nitric oxide (F_{ENO}) has the potential to discriminate between ongoing type-2 airway inflammation or infection in breakthrough attacks on mepolizumab [2].
